# Post-activation depression of the Hoffman reflex is not altered by galvanic vestibular stimulation in healthy subjects

**DOI:** 10.3389/fnint.2023.1234613

**Published:** 2023-08-30

**Authors:** Mónica del Carmen Alvarado-Navarrete, Adriana C. Pliego-Carrillo, Claudia Ivette Ledesma-Ramírez, Carlos A. Cuellar

**Affiliations:** ^1^Biomedical Engineering, School of Medicine, Autonomous University of the State of Mexico, Toluca, Mexico; ^2^School of Sport Sciences, Universidad Anáhuac México, Huixquilucan, Mexico

**Keywords:** H-reflex, post-activation depression, galvanic vestibular stimulation, vestibulospinal pathway, Hoffman (H) reflex

## Abstract

The comprehension of the neural elements interacting in the spinal cord affected by vestibular input will contribute to the understanding of movement execution in normal and pathological conditions. In this context, Hoffman’s reflex (H-reflex) has been used to evaluate transient excitability changes on the spinal cord descending pathways. The post-activation depression (P-AD) of the H-reflex consists of evoking consecutive responses (>1 Hz) provoking an amplitude depression, which has been shown to diminish in pathological conditions (i.e., spasticity, diabetic neuropathy). Galvanic Vestibular Stimulation (GVS) is a non-invasive method that activates the vestibular afferents and has been used to study the excitability of the H-reflex applied as a conditioning pulse. To our knowledge, there are no reports evaluating the P-AD during and after GVS. Our primary aim was to determine if GVS alters the P-AD evoked by stimulating the tibial nerve at 0.1, 1, 5, and 10 Hz, recording in the gastrocnemius and soleus muscles. Direct current stimulation of 2.0 ± 0.6 mA with the cathode ipsilateral (Ipsi) or contralateral (Contra) to the H-reflex electrode montage was applied bilaterally over the mastoid process in 19 healthy subjects. The P-AD’s immediate post-GVS response (P Ipsi, P Contra) was also analyzed. Secondarily, we analyzed the excitability of the H-reflex during GVS. Responses evoked at 0.1 Hz with GVS, post-GVS, and a Control (no GVS) condition were used for comparisons. Our results show that P-AD persisted in all subjects despite increased excitability induced by GVS: statistical significance was found when comparing P-AD at 1, 5, and 10 Hz with the corresponding condition (Control, Ipsi, P Ipsi, Contra, P Contra) at 0.1 Hz (*p* < 0.001). Additionally, the increase in excitability produced by GVS was quantified for the first H-reflex of each P-AD stimulation frequency. The percentage change for all GVS conditions surpassed the Control by at least 20%, being statistically significant for Contra compared to Control (*p* < 0.01). In summary, although GVS increases the excitability of the vestibulospinal pathway at a premotor level, the neural inhibitory mechanism present in P-AD remains unaltered in healthy subjects.

## 1. Introduction

Postural adjustments result from the complex coactivity of the vestibular organs (otoliths and semicircular canals), cerebellar activity, and antigravity muscle proprioceptors (Sugita et al., 2004; Kitajima et al., 2006). Projections between vestibular nuclei and the spinal cord integrate neural responses that maintain balance control (Peusner et al., 2012). The lateral, medial and spinal vestibular nuclei project to the spinal cord through the vestibulospinal tract (VST). The VST is divided into two pathways: the lateral and medial vestibulospinal tracts (LVST and MVST, respectively). Particularly, the LVST provides inputs to the forelimb and hindlimb Rexed’s lamina VII and VIII, exerting excitation to extensor motor neurons and inhibition to flexor motor neurons, thus integrating vestibulospinal reflexes (VSR) (Shinoda et al., 1986).

Galvanic Vestibular Stimulation (GVS) is a non-invasive method that modulates the vestibular system firing activity of the semicircular canals and the otolith organs (Wardman et al., 2003). The influence of GVS on LVST can be studied by performing H-reflex recordings in the lower limbs. Typically, stimulation protocols use GVS as a conditioning pulse to modify H-reflex amplitude. For example, GVS applied 100 ms before H-reflex stimulation in 10 healthy subjects provoked a modulation effect with anodal and cathodal stimulation, decreasing and increasing H-reflex amplitude, respectively (Kennedy and Inglis, 2001). In another study, the H-reflex amplitude recorded 2 min after GVS anodal current decreased as compared to the control condition (Čobeljić et al., 2016). Matsugi et al. (2017) found that H-reflex facilitation induced by GVS increased after only 5 min of performing Gaze Stabilization Exercise (GSE) (Matsugi et al., 2017). Additional maneuvers have been added to evaluate the effect of GVS on the H-reflex, for example, it was found that posture influences H-reflex amplitude facilitated by GVS (Tanaka et al., 2021). The head position must also be considered while assessing H-reflex amplitude (Okada et al., 2018; Nepveu et al., 2020). In 10 healthy subjects, Kennedy and Inglis (2002) found that H-reflex modulation by GVS showed a larger response with anodal stimulation and subjects turning to the right, and cathodal stimulation with subjects turning to the left, than any other polarity or head position combination (Kennedy and Inglis, 2002).

By combining GVS and Transcranial Magnetic Stimulation (TMS), Sayenko et al. (2018) studied the effect of both stimuli on spinally evoked motor potentials (SEMPs). Although GVS and TMS showed a facilitator effect on SEMPs, the conditioning effects lasted longer with GVS (>200 ms) (Sayenko et al., 2018). Another study used GVS and TMS as vestibular and corticospinal input, respectively, to determine its influence at premotor (H-reflex) and motoneuron (electromyography) levels by analyzing amplitude and latency. The results suggested that modulation with GVS occurs at premotor neuron and motor neuron levels but is not influenced by cortical activity (Nepveu et al., 2020). Moreover, cerebellar repetitive TMS followed by noisy GVS facilitated the H-reflex ratio excitability recorded 100 ms after a vestibulospinal function test performed with squared GVS in healthy adults. The combination of these stimulation techniques contributed to inferring the cerebellar influence on vestibulospinal activity (Matsugi et al., 2020) and the vestibular modulatory activity on cervical interneurons by studying myogenic evoked potentials amplitude recorded at the biceps brachii muscles (Suzuki et al., 2017).

The P-AD of the H-reflex (P-AD hereinafter) is a well-documented phenomenon in animals and humans. The P-AD occurs when a peripheral nerve is electrically stimulated at frequencies >1 Hz, consequently, the amplitude of the following H-reflexes recorded at a selected muscle decreases as compared to the first one (Ishikawa et al., 1966; Crone and Nielsen, 1989; Hultborn et al., 1996). Although the mechanisms of P-AD are not fully understood, results in healthy subjects indicate that both presynaptic and postsynaptic mechanisms contribute to this phenomenon (Özyurt et al., 2020).

To our knowledge, there are no reports evaluating the effect of cathodal and anodal GVS on the P-AD. We hypothesized that, as GVS modulates the excitability at the premotor and motor neuron levels by changing the amplitude of the H-reflex, an altered, but not suppressed P-AD would occur provoked by the combination of stimuli. Excitability alterations have been seen previously when combining cutaneous stimulation with cathodal and anodal GVS (Lowrey and Bent, 2009). The main aim of this study was to evaluate the P-AD in the lower limbs before, during, and after cathodal and anodal bilateral GVS in healthy subjects. The secondary aim was to evaluate the excitability of the H-reflex during bilateral GVS.

## 2. Materials and methods

This descriptive, cross-sectional study was performed on 19 healthy volunteers (9 women, 10 men) after an initial screening of 26 subjects (13 women, 13 men). The sample means for age, height, and weight were 22 ± 2 years, 165.6 ± 7.9 cm, and 62.9 ± 13.4 kg, respectively. All subjects showed blood pressure normotensive values (systolic 110 ± 12 mmHg, diastolic 70 ± 8 mmHg). Enrolled individuals reported no history of auditory, vestibular, neurodegenerative, or cardiovascular disease; no subject was under medical treatment. The research protocol was approved by the Faculty of Medicine Ethics in Research Committee of the Autonomous University of the State of Mexico (January 2022, Registration No. CEI/001/2022). Subjects signed an Informed Consent before the experimental session.

### 2.1. H-reflex

**FIGURE 1:**
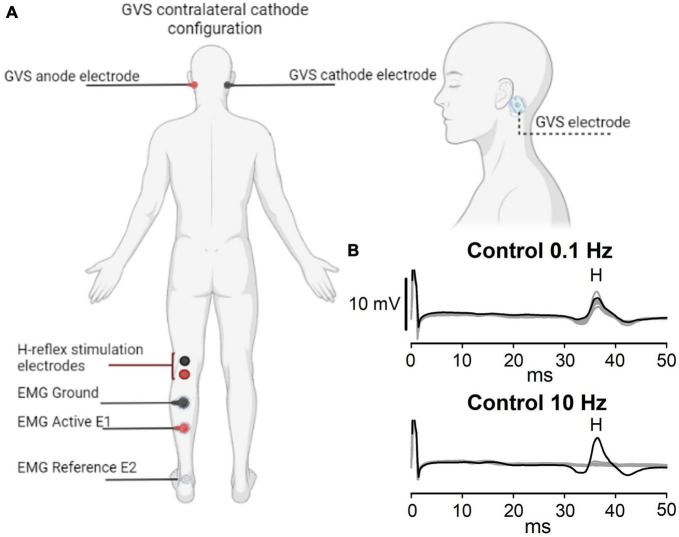
Experimental setup. **(A)** GVS and H-reflex electrode montage. The cathode is ipsilateral (Ipsi) or contralateral (Contra) to the H-reflex recording. **(B)** A representative example of the P-AD evoked at 10 Hz is shown in the lower panel, while stimulation at 0.1 Hz is shown in the upper panel. Black lines represent the first H-reflex; gray lines represent the following nine responses. Note that consecutive H-reflexes (H_2_, H_3_…H_10_) are suppressed, i.e., the P-AD at 10 Hz, but not at 0.1 Hz. Letter H in panel **(B)** represents the appearance of the H-reflex.

The H-reflex was evoked in the left hindlimb by stimulating the tibial nerve and recorded in the soleus muscle (Galvez-Jimenez et al., 2021) as shown in Figure 1A. Before placing the stimulating and recording electrodes, the skin was rubbed with isopropyl alcohol and abraded. Monophasic 1 ms square pulses were applied (Digitimer D8SR, Welwyn Garden City, UK) over the fossa popliteal with a bar electrode (MF Medical, Duiven, Netherlands). The H-reflex was recorded with disposable surface electrodes (Red Dot™ 3M, Saint Paul, MN, USA), digitalized (PowerLab 8/35, AD Instruments, Dunedin, New Zealand), and stored for offline analysis. Initially, pulses were applied at 0.1 Hz in 0.5 mA steps starting at 1 mA. Once consistent H-reflex response was visualized, the bar electrode was fastened and kept in the same anatomic location during the protocol. Then, the current intensity vs. amplitude curves of the H-reflex were recorded. The current intensity was determined from the ascending limb of the recruitment curve at a value corresponding to approximately 40% of the maximal amplitude of the H-reflex and was used for the rest of the protocol, including the P-AD. Typically, the P-AD phenomenon is produced by paired-pulse stimulation. However, it was recently described the importance of evoking >2 consecutive responses, as the second H-reflex may not represent the maximal depression from the first one when comparing only two evoked potentials (Worthington et al., 2021). For this reason, ten pulses (identified by H_1_, H_2_…H_10_) were delivered at 0.1, 1, 5, and 10 Hz in a random sequence. A 0.1 Hz stimulation frequency was used as a reference to compare P-AD evoked at 1, 5, and 10 Hz. The P-AD protocol was applied before (i.e., no GVS, referred to as Control), during, and immediately after (<1 min) anodal or cathodal GVS (P Contra, P Ipsi) for all stimulation frequencies.

### 2.2. GVS

Disposable surface electrodes (Red Dot™ 3M, Saint Paul MN, USA) were placed bilaterally over the mastoid process of all subjects after skin abrasion (Figure 1A). Direct current stimulation was incremented in steps of 0.2 mA from 0.5 mA to a maximum tolerable value without experiencing pain (Barthélemy et al., 2015). The same GVS intensity was applied to the experimental maneuvers. The mean GVS applied with cathodal and anodal currents was 2.0 ± 0.6 mA.

### 2.3. Experimental protocol

Subjects were recumbent in a prone position and asked to face forward, with the head supported by a pillow to avoid a forced extension of the neck. Figure 1A shows the GVS and H-reflex electrode placement. To illustrate the P-AD, representative traces are shown in Figure 1B. At 0.1 Hz, the amplitude of 10 consecutive pulses (gray lines) did not decrease significantly as compared to the first pulse (black lines). In contrast, at 10 Hz, a marked depression of H-reflexes occurred in traces 2–10 (gray traces).

**FIGURE 2:**
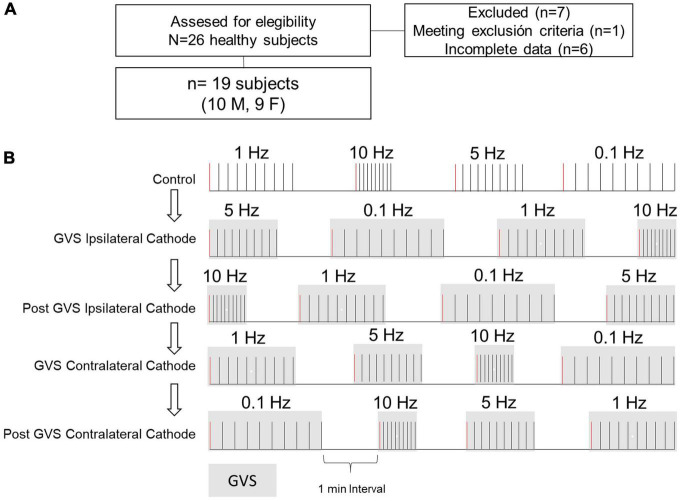
Participants selection and protocol outline. **(A)** Participants eligibility criteria. **(B)** The P-AD at 1, 5, and 10 Hz was evaluated before (Control, i.e., no GVS), during (Ipsi, Contra), and after (P Ipsi, P Contra) GVS. For comparison, H-reflex with the same stimulation conditions at 0.1 Hz were analyzed. The diagram exemplifies the delivery of 10 consecutive pulses to evaluate P-AD with randomization of stimulation frequencies for each subject. Gray squares represent GVS application. First H-reflex (red lines) represents the responses used to evaluate changes in excitability in the H-reflex. For example, for each subject, four H-reflexes were averaged in each condition.

The P-AD protocol was applied before (Control), during (Ipsi, Contra), and after GVS (P Ipsi, P Contra), with the cathode ipsilateral (Ipsi) or contralateral (Contra) to the left leg of the H-reflex recording (Figure 1A) in one session. The experimental protocol and eligibility criteria are outlined in Figures 2A, B. The lower diagram (Figure 2B) shows the order of maneuvers and an example of how stimulation frequencies (ten pulses at 0.1, 1 Hz, and 10 Hz) to evoke P-AD were applied during GVS (gray squares).

### 2.4. Data analysis

Recordings of the H-reflexes were segmented in 50 ms windows starting from the stimulus artifact as seen in Figure 1B. Peak amplitude (PA) and area under the curve (AUC) were calculated for each potential. AUC of H-reflexes was used to obtain the ratios (H_2_/H_1_, H_3_/H_1_…H_10_/H_1_) AUC to represent the P-AD. Then, the mean P-AD was determined as the average of the AUC ratios [(H_2_/H_1_ + H_3_/H_1_ + … + H_10_/H_1_)/9]. To determine changes in the excitability of the H-reflex, the PA of the first H-reflex (H_1_) in each stimulation frequency (0.1, 1, 5, and 10 Hz) was pooled and averaged, then the percentage of change of the H-reflex during (Ipsi, Contra) and after (P Ipsi, P Contra) GVS was obtained and compared to Control per subject (i.e., each condition is composed by 4 H-reflexes H_1_). The excitability analysis of P-AD with GVS was determined by averaging the PAs of H_2_-H_10_ for each stimulation frequency; then the mean values in each GVS condition (Ipsi, P Ipsi, Contra, P Contra) were normalized to the corresponding mean of the Control condition.

To determine if GVS altered the amplitude variability of the H-reflex and P-AD, the variation coefficient (VC) was estimated from the mean and standard deviation (SD) values of the first potential detected for all frequencies (H-reflex, H_1_) and for the mean and SD values of the normalized potentials (P-AD, H_2_-H_10_) in each GVS condition (Ipsi, P Ipsi, Contra, P Contra).

For statistical comparison, mean and SD values were calculated for PA, AUC, P-AD, and VC, before, during (Ipsi, Contra), and after GVS (P Ipsi, P Contra) for each stimulation frequency. The effect of polarity of the stimulus, during and after GVS was also considered in Ipsi/Contra vs P Contra/P Ipsi comparisons. The normality of data was tested with the Shapiro–Wilk test, and if *p* > 0.05, statistical differences between groups were tested with the one-way repeated measures ANOVA. If normality failed, Friedman Test was used instead. Tukey’s test was used for *post hoc* analysis. Significant differences were established if *p* < 0.05.

## 3. Results

### 3.1. Effect of GVS on the P-AD

**FIGURE 3:**
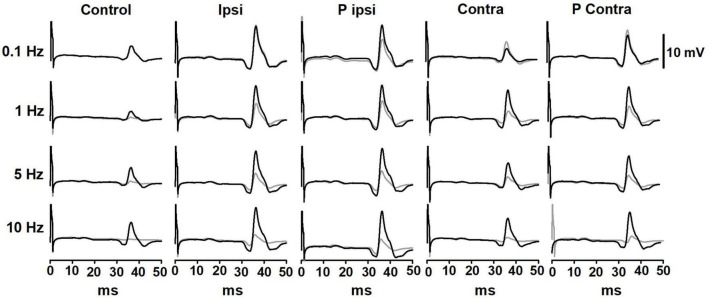
Representative traces of the P-AD before (Control), during (Ipsi and Contra), and after (P Ipsi and P contra) GVS in one subject. For each tested condition, in black, 1st H-reflex; in gray, mean trace from 2nd to 10th H-reflexes. As a comparison, stimulation frequency at 0.1 Hz is also shown in all GVS conditions. GVS increases the H-reflex amplitude in all stimulation frequencies but does not interfere notably with the P-AD. The excitability increase is present also in P Ipsi and P Contra.

A representative example of the effect of GVS on the P-AD in a subject is shown in Figure 3. Black lines represent the 1st H-reflex while gray lines represent the average of 2nd–10th responses. Note that during stimulation at 0.1 Hz, there is no P-AD in any GVS condition. In contrast, P-AD was observed at 1, 5, and 10 Hz, during cathode ipsilateral (Ipsi) and cathode contralateral (Contra) to H-reflex recording. Note that compared to Control, an increase in the amplitude of H-reflexes occurred during (Ipsi and Contra) and after (P Ipsi and P Contra) GVS conditions (Figure 3). An overall increase in the excitability of evoked responses during (Ipsi, Contra) and after GVS (P Ipsi, P Contra) was observed compared to Control in all stimulation conditions and stimulation frequencies, this is, the mean amplitude of the 2nd–10th H-reflexes increased compared to the Control (No GVS).

**FIGURE 4:**
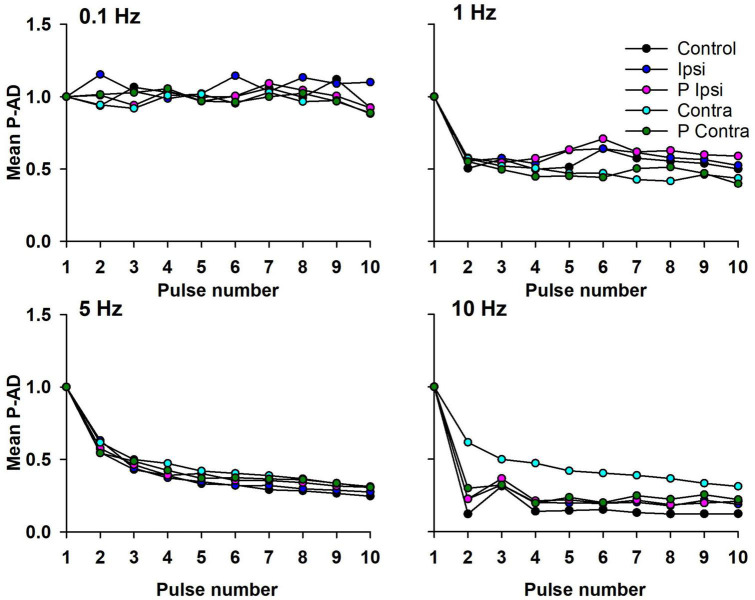
Mean AUC of the P-AD plotted by pulse number (H_2_/H_1_…H_10_/H_1_) in all GVS conditions (Ipsi, Contra, P Ipsi, P Contra). As a reference, 0.1 Hz stimulation frequency was plotted (*n* = 19). SD is not shown for clarity purposes.

To evaluate the pulse-by-pulse behavior of the P-AD across GVS conditions, the mean AUC ratios (H_1_/H_1_, H_2_/H_1_, H_3_/H_1_…H_10_/H_1_) vs. pulse number were plotted as shown in Figure 4 (*n* = 19). At 0.1 Hz there are no noticeable changes in the amplitude of the H-reflex in the Control condition (black circles) nor during (Ipsi, blue circles/Contra, cyan circles) and post-GVS (P Ipsi, magenta circles/P Contra, green circles). On the contrary, P-AD is characterized by a pronounced decrease in the AUC amplitude of consecutive H-reflexes compared to the first. The depression is more evident as the stimulation frequency increases (≈60% at 1 Hz, ≈40% at 5 Hz and ≈30% at 10 Hz) (Figure 4). It can be noticed that P-AD is preserved across all GVS (Ipsi, Contra) and post-GVS (P Ipsi, P Contra) conditions at all stimulation frequencies (1, 5, and 10 Hz).

**FIGURE 5:**
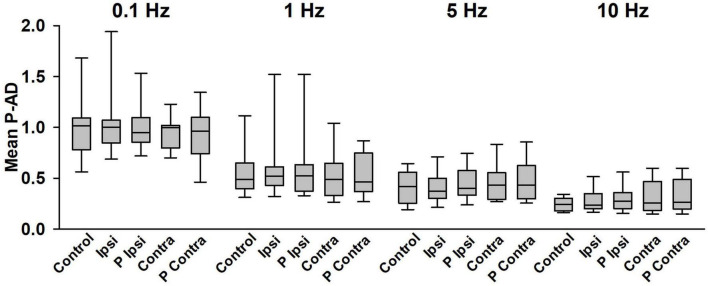
Mean P-AD is not affected by GVS at any stimulation frequency. No statistical differences were found between any GVS condition vs. Control in each stimulation frequency (One Way Repeated Measures ANOVA). Statistical differences were found between Control, Ipsi, P Ipsi, Contra, P Contra at 0.1 Hz vs the corresponding condition at all stimulation frequencies shown in Table 1, *n* = 19 (One Way Repeated Measures ANOVA, *post hoc* Hold-Sidak Method). Statistical differences are not shown in the figure for clarity purposes.

The mean of the P-AD [(H_2_/H_1_ + H_3_/H_1_ + … + H_10_/H_1_)/9] for each stimulation frequency is shown in Figure 5 and in Table 1 for all tested conditions (*n* = 19). At 0.1 Hz, Ipsi, P Ipsi, Contra and P Contra did not show statistical differences compared to Control (*p* > 0.05). Similarly, mean P-AD at 1, 5 and 10 Hz (Ipsi, P Ipsi, Contra and P Contra) were compared against their corresponding Control; no statistical differences were found (*p* > 0.05). This means that during and after GVS (Ipsi, P Ipsi, Contra and P Contra), P-AD is not affected at any stimulation frequency. Then, mean P-AD at 1, 5, and 10 Hz at Control, Ipsi, P Ipsi, Contra and P Contra were compared to the correspondent Control or GVS condition at 0.1 Hz. Statistical differences were found in all P-AD frequencies and GVS configurations (Figure 5; Table 1), indicating that P-AD is preserved and is not significantly affected by GVS.

**TABLE 1:** Mean P-AD in all GVS conditions and stimulation frequencies.

Stim Freq (Hz)	Control	GVS cathode ipsilateral	Post GVS cathode ipsilateral	GVS cathode contralateral	Post GVS cathode contralateral
0.1	**1.01 ± 0.32**	**1.03 ± 0.45**	**0.96 ± 0.39**	**0.96 ± 0.19**	**0.92 ± 0.29**
1	0.58 ± 0.41 *p* < 0.001	0.6 ± 0.36 *p* < 0.001	0.64 ± 0.42 *p* < 0.001	0.55 ± 0.31 *p* < 0.001	0.52 ± 0.21 *p* < 0.001
5	0.40 ± 0.17 *p* < 0.001	0.42 ± 0.20 *p* < 0.001	0.45 ± 0.18 *p* < 0.001	0.44 ± 0.17 *p* < 0.001	0.48 ± 0.22 *p* < 0.001
10	0.26 ± 0.15 *p* < 0.001	0.28 ± 0.14 *p* < 0.001	0.29 ± 0.27 *p* < 0.001	0.32 ± 0.17 *p* < 0.001	0.33 ± 0.18 *p* < 0.001

Pair-wise comparisons using the Holm–Sidak method. All GVS conditions during stimulation frequencies were compared to their corresponding Control or GVS condition at 0.1 Hz (bold numbers).

### 3.2. Effects of GVS on the H-reflex

**FIGURE 6:**
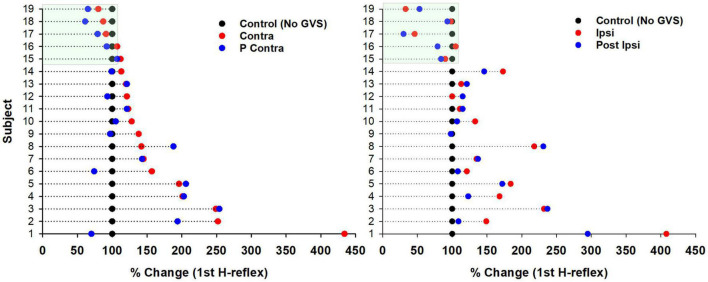
Changes in excitability of the H-reflex produced by GVS per subject. Percentage of change of the H-reflex in each GVS condition (Contra, P Contra, **left panel**; Ipsi, P Ipsi, **right panel**) compared to Control per subject (*n* = 19). Each dot represents the average of the first H-reflex at 0.1, 1, 5, and 10 Hz (four H-reflexes per subject) for each GVS condition normalized to the average Control (100%).

Although we did not observe significant changes in the P-AD produced by GVS, we observed changes in excitability in the H-reflex PA (1st response in each stimulation frequency, PA, see Section “2. Materials and methods”) compared to Control. Results are presented as percentage of change per subject in Figure 6 (*n* = 19). An increase in excitability occurred in 16 subjects in Contra, in 9 subjects in P Contra, in 12 subjects in Ipsi, and in 13 subjects in P Ipsi. On the contrary, the H-reflex PA decreased in 3 subjects in Contra and in 4 subjects in P Contra. A decrease was also observed in 4 subjects during Ipsi and in 5 subjects in P Ipsi. Overall, GVS increased the excitability of the H-reflex regardless of the stimulation polarity (Ipsi, Contra) and after GVS (P Ipsi, P Contra).

**FIGURE 7:**
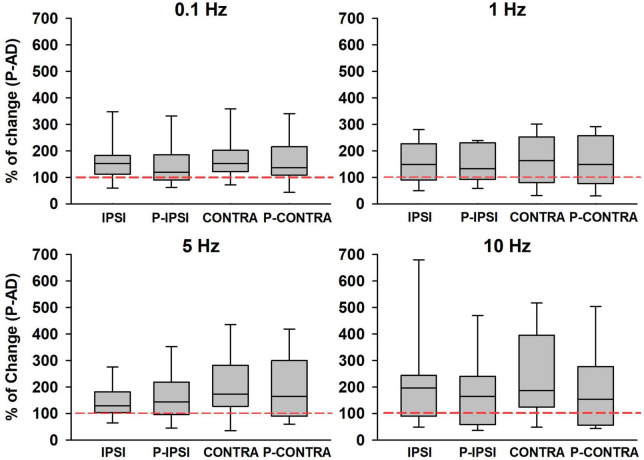
An increase in the PA of the averaged H-reflexes (2nd–10th) during the P-AD was observed in all GVS conditions (Ipsi, P Ipsi, Contra, P Contra) normalized to the Control mean (red dotted line), *n* = 19. No statistical difference of normalized PA was found between conditions for any P-AD stimulation frequency (One Way Repeated Measures ANOVA, *post hoc* Holm–Sidak Method).

Then, we analyzed if GVS produced changes in excitability during the P-AD (Figure 7). For this purpose, the mean PA of the 2nd–10th pulses were normalized, the Control mean is represented as 100% (red dotted line) for each stimulation frequency, boxplots correspond to GVS conditions (Ipsi, Contra, P Ipsi, P Contra) (Figure 7). Results are presented as percentage of change for all subjects (*n* = 19). An overall increase in excitability (>100%) was observed in all GVS conditions (Ipsi, Contra, P Ipsi, P Contra) and stimulation frequencies. However, no statistical difference was found between GVS conditions in any P-AD frequency tested (Table 2).

**TABLE 2:** Mean percentage change of ± SD normalized to mean Control (100%) for each GVS condition and P-AD stimulation frequency.

Stim Freq (Hz)	GVS ipsilateral cathode (Ipsi)	Post GVS ipsilateral cathode (P Ipsi)	GVS contralateral cathode (Contra)	Post GVS contralateral cathode (P Contra)
0.1	171.7 ± 103.1	160.10 ± 123.1	177.5 ± 100.8	163.5 ± 86.4
1	156.8 ± 100.2	155.9 ± 113.0	157.4 ± 87.9	150.9 ± 95.1
5	140.4 ± 89.8	143.7 ± 101.7	178.6 ± 109.0	175.8 ± 115.6
10	195.2 ± 147.9	166.7 ± 121.9	197.9 ± 127.2	167.9 ± 127.3

No statistical differences were found between GVS conditions in any stimulation frequency.

However, PA without normalization showed a significant increase when comparing Control (Control 4.24 ± 3.78 μV) vs. Ipsi (6.20 ± 4.42 μV) *p* < 0.001, P Ipsi (5.84 ± 4.33 μV) *p* < 0.01, Contra (6.25 ± 4.52 μV) *p* < 0.001, and P Contra (6.24 ± 4.73 μV) *p* < 0.001 at 0.1 Hz; between Control (1.75 ± 1.91 μV) vs. Contra (3.01 ± 2.81 μV) *p* < 0.05, and P Contra (3.06 ± 3.00 μV) *p* < 0.05 at 5 Hz; and between Control (0.73 ± 0.56) and Contra (1.82 ± 2.21 μV) *p* < 0.05 at 10 Hz.

**FIGURE 8:**
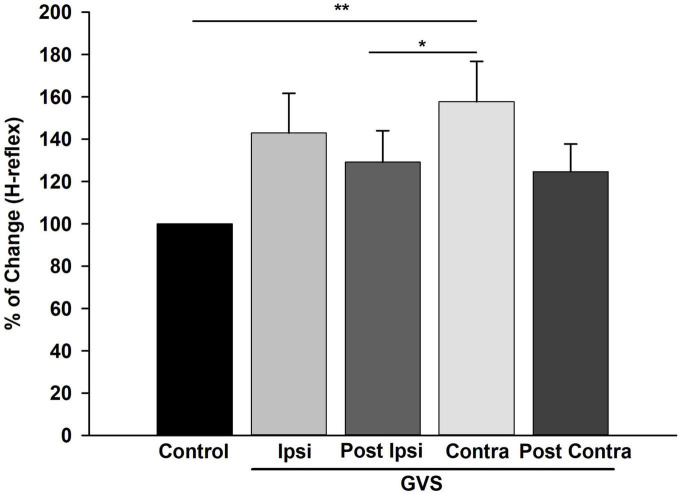
Galvanic Vestibular Stimulation (GVS) produced an increase in excitability in the H-reflex. Mean of the percentage of change of the H-reflex in each GVS condition (Ipsi, P Ipsi, Contra P Contra) compared to Control. Each bar represents the average of the first H-reflex in each stimulation condition (0.1, 1, 5, and 10 Hz) normalized to Control (*n* = 19). **p < 0.01, *p < 0.05 (Friedman Repeated Measures, ANOVA on Ranks, *post-hoc* Tukey Test). GVS produced an increase in the amplitude of the H-reflex statistically significant for the Contra condition compared to Control.

Next, the amplitude of the first H-reflex for all stimulation frequencies was pooled and averaged to determine the percentage of change of the H-reflex across GVS conditions compared to Control. The percentage of change mean of the H-reflex across GVS conditions is shown in Figure 8. Statistical differences between Control vs. Contra (*p* = 0.002), and Contra vs. P Ipsi (*p* < 0.04) were found. In summary, Figure 8 indicates that GVS increases the excitability of the H-reflex in all conditions, as also shown in Figures 6, 7. The post-GVS conditions show a slight decrease in the percentage of change compared to the GVS condition (Ipsi, Contra) without fully returning to the Control values.

We also determined VC by two parameters: PA and AUC. The analysis of the first H-reflex (H_1_) mean VC for each stimulation frequency in each GVS condition shows that PA and AUC variability decreases with GVS (PA: Control 33.56 ± 24.08%, Contra 27.55 ± 37.13%, P Contra 18.95 ± 15.82%, Ipsi 19.80 ± 16.39%, P Ipsi 21.02 ± 19.33%; AUC: Control 31.79 ± 21.51%, Contra 18.55 ± 18.25%, P Contra 21.59 ± 20.85%, Ipsi 21.05 ± 17.99%, P Ipsi 23.01 ± 19.81%). Variability of the PA and AUC increases with P-AD stimulation frequency as compared to the correspondent Control or GVS condition at 0.1 Hz, however, when comparing VC changes in each stimulation frequency, no clear pattern appears to establish how GVS influences changes in variability (Table 3).

**TABLE 3:** Mean variation coefficient of PA (μV) and AUC (μV^2^) ± SD for each GVS condition and P-AD stimulation frequency.

Stim Freq (Hz)	Control	GVS ipsilateral cathode (Ipsi)	Post GVS ipsilateral cathode (P Ipsi)	GVS contralateral cathode (Contra)	Post GVS contralateral cathode (P Contra)
0.1	PA: 9.37 ± 13.84 AUC: 19.14 ± 13.05	PA: 14.04 ± 10.73 AUC: 16.29 ± 12.25	PA: 18.23 ± 13.21 AUC: 19.94 ± 16.68	PA: 16.93 ± 17.65 AUC: 13.60 ± 90.94	PA: 16.56 ± 12.96 AUC: 17.01 ± 13.41
1	PA: 28.61 ± 11.10 AUC: 28.90 ± 12.17	PA: 29.20 ± 15.27 AUC: 29.24 ± 14.27	PA: 27.59 ± 16.32 AUC: 26.23 ± 13.77	PA: 23.18 ± 14.78 AUC: 26.03 ± 19.01	PA: 24.89 ± 15.56 AUC: 24.07 ± 14.25
5	PA: 35.74 ± 21.61 AUC: 39.13 ± 26.74	PA: 46.32 ± 27.49 AUC: 43.10 ± 24.91	PA: 38.88 ± 24.76 AUC: 35.33 ± 24.72	PA: 35.29 ± 22.02 AUC: 31.81 ± 20.13	PA: 32.94 ± 20.86 AUC: 30.76 ± 22.06
10	PA: 62.15 ± 33.40 AUC: 61.84 ± 37.93	PA: 42.10 ± 22.04 AUC: 41.47 ± 19.95	PA: 45.57 ± 29.76 AUC: 43.90 ± 26.64	PA: 53.92 ± 36.79 AUC: 54.40 ± 37.06	PA: 55.92 ± 41.02 AUC: 51.42 ± 39.64

Finally, the latency of H-reflexes was not significantly affected by GVS at any stimulation frequency: 0.1 Hz: Control, (30.97 ± 2.06 ms); Ipsi (30.92 ± 1.88 ms); P Ipsi (31.00 ± 2.22 ms); Contra (31.36 ± 2.12 ms); P Contra (31.28 ± 2.24 ms); 1 Hz, Control, (30.89 ± 2.03 ms); Ipsi (30.76 ± 1.91 ms); P Ipsi (30.82 ± 2.08 ms); Contra (30.62 ± 2.08 ms), P Contra (30.92 ± 2.03 ms); 5 Hz, Control, (30.79 ± 1.90 ms), Ipsi (30.41 ± 2.07 ms); P Ipsi (30.57 ± 2.61 ms); Contra (30.41 ± 2.41 ms), P Contra (30.68 ± 2.01); 10 Hz, Control, (30.44 ± 2.04 ms); Ipsi (30.30 ± 2.31 ms); P Ipsi (30.50 ± 2.57 ms), Contra (30.95 ± 2.34), P Contra (30.39 ± 2.14 ms).

## 4. Discussion

The effect of GVS on the H-reflex in healthy subjects has been studied previously to evaluate the vestibular influence on premotor and motor excitability in the spinal cord. However, no previous research was found evaluating the vestibular influence on H-reflex amplitude, nor in P-AD during GVS. The P-AD phenomenon has been studied typically by paired-pulse stimulation; however, it was recently described the importance of evoking >2 consecutive responses, as the second H-reflex may not represent the maximal depression compared to the first one (Worthington et al., 2021). For this reason, in our protocol, 10 consecutive pulses were applied to characterize the behavior of individual pulses (2nd–10th) and to determine the mean P-AD at 1, 5, and 10 Hz; for comparison, H-reflex was evoked by stimulating at 0.1 Hz.

The mechanisms producing the P-AD are not completely understood. Based on previous findings indicating that orthodromic action potentials generated by Primary Afferent Depolarization and recorded as Dorsal Root Reflexes can produce EPSPs in motoneurons; Hari et al. (2022) and Metz et al. (2023) proposed that the P-AD is produced by a mechanism of presynaptic inhibition (PSI). The authors proposed that the orthodromic potentials invading the Ia terminal and generated by the facilitation of sodium spikes via GABA-A receptors near Ranvier nodes, could reduce neurotransmitter release long enough to decrease the amplitude of subsequent H-reflexes and that postsynaptic inhibition mediated by GABA on GABA-A and GABA-B receptors on motoneurons may account for long depression during the P-AD. Figure 11 in Metz et al. (2023) depicts the circuits mediating P-AD through primary afferent depolarization evoked spikes that travels toward the Ia afferent terminal, and to glutamatergic neurons that activate GABAergic neurons and consequently GABA-B receptors in the same Ia afferent. Although additional information is necessary, this scenario shows multiple PSI mechanisms. In this context, by applying a sustained stimulation over the mastoid process while evoking P-AD, it was certain that the changes in excitability produced by GVS would be present at all tested frequencies of P-AD, including the stimulation frequency at 0.1 Hz. For instance, in our protocol, the longest period with GVS at 0.1 Hz lasted 100 s (10 s × 10 repetitions), while the longest period of excitability increase produced by conditioning pulses found in the literature was 300 ms (Sayenko et al., 2018).

In this study, the effect of GVS on the P-AD was described for the first time. We found that the P-AD was not significantly affected during (Ipsi, Contra) and post-GVS (P Ipsi, P Contra) conditions compared to Control, i.e., the mean AUC decrease from 2nd to 10th H-reflex potentials was ≈ 60, 40, and 30%, at 1, 5, and 10 Hz stimulation frequencies, respectively, showing that P-AD was preserved (Figures 4, 5). As expected, an overall increase in the amplitude of individual H-reflexes was observed during (Ipsi/Contra) and post-GVS (P Ipsi/P Contra) in all stimulation frequencies compared to control (Figures 6–8), however, no previous study was found analyzing H-reflex response during GVS. Additionally, we analyzed the changes in variability provoked by GVS on the PA and AUC H-Reflex, and the P-AD. Interestingly, H-reflex (first response pooled for all stimulation frequencies) variability decreased during and after GVS, while P-AD variability incremented with stimulation frequency, although no clear effect of GVS was identified.

Overall, our results confirm previous findings indicating that GVS increases the excitability at the neural elements involved in the H-reflex (Lowrey and Bent, 2009; Okada et al., 2018; Nakamura et al., 2021); however, this increase in excitability did not affect the P-AD in healthy individuals, suggesting preservation of inhibitory spinal mechanisms. In this context, it has been described that a reduction or loss of the P-AD in pathological conditions occurs, such as in multiple sclerosis (Cantrell et al., 2022), spinal cord injury (Nielsen et al., 1995; Knikou and Murray, 2019), and recently, in diabetic neuropathy (Lee-Kubli and Calcutt, 2014; Marshall et al., 2017; Worthington et al., 2021) and prediabetic, overweight and obese subjects (Salinas et al., 2022).

It is noticeable the high variability of PA and AUC in P-AD, so its origin must be discussed to understand our findings. Regarding GVS, variability has been attributed to experimental characteristics such as current intensity and polarity (Nakamura et al., 2015; Sprenger et al., 2020), electrode montage (bilateral or unilateral) (Pliego et al., 2021; Tanaka et al., 2021), body posture (Iles et al., 2007; Hannan et al., 2021), and neck position (Osler and Reynolds, 2012). The current intensity is associated with the modified level of excitability, with the most notable amplitude increase observed in H-reflex amplitude using a conditioning GVS pulse between 2–3 mA (Okada et al., 2018). Our results show an amplitude increase of the H-reflex using a stimulation intensity in the range of 2.0 ± 0.6 mA, so we may assume that the changes in excitability are equivalent to those previously reported with larger current intensities (Lowrey and Bent, 2009; Okada et al., 2018; Tanaka et al., 2021).

The origin of H-reflex variability must also be considered. First, the individual cutaneous, motor, and movement perception thresholds using GVS activate separate nervous pathways, thus, provoking dissimilar effects on excitability (Wuehr et al., 2018; Mikhail et al., 2021). Another important factor about H-reflex is that variability increases at the intensity where both M and H waves are present, stressing the need for PA and AUC normalization (Brinkworth et al., 2007). Additional variability elements are diverse, going from body posture, plantar flexion, electrode position, and current intensity, to the excitability state of the motor pool at a particular time of the muscle contraction (Funase and Miles, 1999). Variability is present depending on the recorded muscles (vastus medialis, lateralis, and rectus femoris), neck position, and eyes closed or open (Kameyama et al., 1989). Although our subjects were indicated to lie prone, facing forward, with eyes open, no restraining device was provided to prevent head movement, allowing eyes opening or closing without supervision. We do not discard other variability sources such as differences in anatomical features related to electrode montage or individual body composition (Bolfe et al., 2007). Our results show that H-reflex PA decreased in 4 subjects (2 females, 2 males) with GVS (Ipsi, Contra) (Figure 6). Nevertheless, after analyzing the participants’ records, we could not identify a possible clinical or anthropometrical parameter causing this result, for example, Body Mass Index (BMI) was in the normal range (18.5–25 kg/m^2^). However, a more profound analysis of the impact of metabolic parameters or preclinical conditions on motor function could determine excitability alterations of H-reflex using GVS.

It is presumed that, while the cathodal electrode has a depolarizing effect on the underlying structures, the anodal electrode is hyperpolarizing. However, we found that both, cathodal and anodal stimulation provoked H-reflex facilitation. This increase in excitability was also observed in a previous study using short interstimulus intervals (Lowrey and Bent, 2009). In addition, anodal current of 8 mA showed no inhibitory effect on short-latency and long-latency electromyographic response of the soleus muscle (Bacsi and Colebatch, 2003). Our finding appears to be in accordance with what has been described extensively at the postural level, for we found a more pronounced increase in excitability with Contra stimulation, this is, with the cathode contralateral to the H-reflex stimulation site, meaning anodal GVS current. Similarly, during walking or standing, the subject veers toward the anode (left or right) with GVS, instead of moving forward, even while following a visual target (Fitzpatrick et al., 1999; Bent et al., 2004; Reimann et al., 2017). To our knowledge, only one publication showed that GVS polarity did not provoke the opposite effect on a motor task, with subjects lying prone, legs extended, and moving a force platform with plantar flexion. Both anodal and cathodal stimulation increased motor unit activity threshold, recorded with needle electrodes, from 8.7 N to ≈ 10.5 N with GVS (Kennedy et al., 2004). The disparity in the observed responses in posture and motor tasks, compared to H-reflex, points to a separate effect of GVS provoked over premotor neurons and the neuromuscular unit. Under our experimental conditions, we assume that the GVS is activating descending vestibulospinal pathways such as the LVST and reticulospinal tract that affect interneuron circuits mediating motoneuron responses (Kennedy et al., 2004).

Altogether, our study adds evidence to continue exploring the use of GVS as a valuable means to evaluate the integrity of the vestibulospinal pathway, for it has become a useful tool in basic, clinical and rehabilitation research (Tax et al., 2013; Welgampola et al., 2013; Čobeljić et al., 2016; Schniepp et al., 2018). In this context, it would be of great interest to study P-AD in combination with GVS in pathological populations that have previously been shown to respond separately to control subjects using GVS alone, as seen in asymptomatic patients with Human T-lymphotropic virus 1 and patients with the associated myelopathy spastic paraparesis (Matos Cunha et al., 2013). Furthermore, GVS in combination with other stimulation techniques, such as TMS (Matsugi et al., 2020) and transcutaneous spinal cord stimulation (Sayenko et al., 2018) has shown to become a promising intervention to understand the modulation of supraspinal structures on the motor output and to evaluate residual function, particularly after spinal cord injury. In summary, we posit that the use of P-AD and GVS can be applied to evaluate vestibulospinal pathways, in pathological and non-pathological conditions.

## 5. Conclusion

Galvanic Vestibular Stimulation did not produce a significant change in the P-AD as compared to Control in healthy subjects, i.e., for this spinal inhibitory process was preserved in all tested conditions (Ipsi, Contra, P Ipsi, and P Contra). Interestingly, an overall increase of excitability of the H-reflex was observed, being statistically different between Control and Contra. However, this increase was observed in 14/19 subjects when responses were averaged across subjects. GVS concedes to study the influence of the vestibular pathway on the spinal circuitry and evaluate associated phenomenon such as P-AD and H-reflex excitability. Our research adds to the available methods that study the integrity of the vestibulospinal tract and its mechanism, putting forward to extend the test to pathological population with altered neural excitability.

## Data availability statement

The datasets presented in this article are not readily available because they are not in a online database, but are available from the corresponding author on reasonable request. Requests to access the datasets should be directed to CC, carlos.cuellarra@anahuac.mx.

## Ethics statement

The studies involving humans were approved by the School of Medicine Ethics in Research Committee of the Autonomous University of the State of Mexico (CONBIOETICA-15-CEI-002-20210531, No. CEI/001/2022). The studies were conducted in accordance with the local legislation and institutional requirements. The participants provided their written informed consent to participate in this study.

## Author contributions

MA-N, AP-C, and CC organized and performed the experiments and analyzed and interpreted the data. MA-N built the experimental data base. AP-C and CC wrote the manuscript. AP-C, CC, and CL-R contributed to the design and discussion of the study. All authors reviewed and approved the submitted version.

## References

[B1] Bacsi A. M. Colebatch J. G. (2003). Anodal vestibular stimulation does not suppress vestibular reflexes in human subjects. *Exp. Brain Res.* 150 525–528. 10.1007/s00221-003-1489-2 12739094

[B2] Barthélemy D. Willerslev-Olsen M. Lundell H. Biering-Sørensen F. Nielsen J. B. (2015). Assessment of transmission in specific descending pathways in relation to gait and balance following spinal cord injury. *Prog. Brain Res.* 218 79–101. 10.1016/bs.pbr.2014.12.012 25890133

[B3] Bent L. R. Inglis J. T. McFadyen B. J. (2004). When is vestibular information important during walking? *J. Neurophysiol.* 92 1269–1275. 10.1152/jn.01260.2003 15102904

[B4] Bolfe V. Ribas S. Montebelo M. Guirro R. (2007). Comportamento da impedância elétrica dos tecidos biológicos durante estimulação elétrica transcutânea. *Rev. Bras. Fisioter.* 11:2. 10.1590/S1413-35552007000200011

[B5] Brinkworth R. S. A. Tuncer M. Tucker K. J. Jaberzadeh S. Türker K. S. (2007). Standardization of H-reflex analyses. *J. Neurosci. Methods* 162 1–7. 10.1016/j.jneumeth.2006.11.020 17257686

[B6] Cantrell G. S. Lantis D. J. Bemben M. G. Black C. D. Larson D. J. Pardo G. (2022). Relationship between soleus H-reflex asymmetry and postural control in multiple sclerosis. *Disabil. Rehabil.* 44 542–548. 10.1080/09638288.2020.1771779 32525405

[B7] Čobeljić R. Miljković N. Ribarić-Jankes K. Švirtlih L. (2016). A paradigm of galvanic vestibular stimulation diminishes the soleus muscle H-reflex in healthy volunteers. *Spinal Cord* 54 150–153. 10.1038/sc.2015.135 26282493

[B8] Crone C. Nielsen J. (1989). Methodological implications of the post activation depression of the soleus H-reflex in man. *Exp. Brain Res.* 78 28–32. 10.1007/BF00230683 2591515

[B9] Fitzpatrick R. C. Wardman D. L. Taylor J. L. (1999). Effects of galvanic vestibular stimulation during human walking. *J. Physiol.* 517 931–939. 10.1111/j.1469-7793.1999.0931s.x 10358131 PMC2269389

[B10] Funase K. Miles T. S. (1999). Observations on the variability of the H reflex in human soleus. *Muscle Nerve* 22 341–346. 10086894 10.1002/(sici)1097-4598(199903)22:3<341::aid-mus6>3.0.co;2-r

[B11] Galvez-Jimenez N. Morren J. A. Soriano A. Armstrong K. Goldberg M. Gonzalez L. (2021). “Atlas of nerve conduction studies (NCS),” in *Electrodiagnostic medicine*, eds Galvez-Jimenez N. Soriano A. Morren J. A. (Cham: Springer International Publishing), 10.1007/978-3-030-74997-2_2

[B12] Hannan K. B. Todd M. K. Pearson N. J. Forbes P. A. Dakin C. J. (2021). Vestibular attenuation to random-waveform galvanic vestibular stimulation during standing and treadmill walking. *Sci. Rep.* 11:8127. 10.1038/s41598-021-87485-4 33854124 PMC8046779

[B13] Hari K. Lucas-Osma A. M. Metz K. Lin S. Pardell N. Roszko D. A. (2022). GABA facilitates spike propagation through branch points of sensory axons in the spinal cord. *Nat. Neurosci.* 25 1288–1299. 10.1038/s41593-022-01162-x 36163283 PMC10042549

[B14] Hultborn H. Illert M. Nielsen J. Paul A. Ballegaard M. Wiese H. (1996). On the mechanism of the post-activation depression of the H-reflex in human subjects. *Exp. Brain Res.* 108 450–462. 10.1007/BF00227268 8801125

[B15] Iles J. F. Baderin R. Tanner R. Simon A. (2007). Human standing and walking: comparison of the effects of stimulation of the vestibular system. *Exp. Brain Res.* 178 151–166. 10.1007/s00221-006-0721-2 17031681

[B16] Ishikawa K. Ott K. Porter R. W. Stuart D. (1966). Low frequency depression of the H wave in normal and spinal man. *Exp. Neurol.* 15 140–156. 10.1016/0014-4886(66)90039-2 5934660

[B17] Kameyama O. Hayes K. C. Wolfe D. (1989). Methodological considerations contributing to variability of the quadriceps H-Reflex. *Am. J. Phys. Med. Rehabil.* 68 277–282. 10.1097/00002060-198912000-00004 2590515

[B18] Kennedy P. M. Cresswell A. G. Chua R. Inglis J. T. (2004). Galvanic vestibular stimulation alters the onset of motor unit discharge. *Muscle Nerve* 30 188–194. 10.1002/mus.20074 15266634

[B19] Kennedy P. M. Inglis J. T. (2001). Modulation of the soleus H-reflex in prone human subjects using galvanic vestibular stimulation. *Clin. Neurophysiol.* 112 2159–2163. 10.1016/S1388-2457(01)00665-4 11682356

[B20] Kennedy P. M. Inglis J. T. (2002). Interaction effects of galvanic vestibular stimulation and head position on the soleus H reflex in humans. *Clin. Neurophysiol.* 113 1709–1714. 10.1016/S1388-2457(02)00238-9 12417223

[B21] Kitajima N. Sugita-Kitajima A. Bai R. Sasaki M. Sato H. Imagawa M. (2006). Axonal pathways and projection levels of anterior semicircular canal nerve-activated vestibulospinal neurons in cats. *Neuroscience Letters* 406 1–5. 10.1016/j.neulet.2006.06.024 16908100

[B22] Knikou M. Murray L. M. (2019). Repeated transspinal stimulation decreases soleus H-reflex excitability and restores spinal inhibition in human spinal cord injury. *PLoS One* 14:e0223135. 10.1371/journal.pone.0223135 31557238 PMC6762874

[B23] Lee-Kubli C. A. G. Calcutt N. A. (2014). Altered rate-dependent depression of the spinal H-reflex as an indicator of spinal disinhibition in models of neuropathic pain. *Pain* 155 250–260. 10.1016/j.pain.2013.10.001 24103402 PMC3946970

[B24] Lowrey C. R. Bent L. R. (2009). Modulation of the soleus H-reflex following galvanic vestibular stimulation and cutaneous stimulation in prone human subjects: H-Reflex after vestibular and cutaneous stimulation. *Muscle Nerve* 40 213–220. 10.1002/mus.21275 19367637

[B25] Marshall A. G. Lee-Kubli C. Azmi S. Zhang M. Ferdousi M. Mixcoatl-Zecuatl T. (2017). Spinal disinhibition in experimental and clinical painful diabetic neuropathy. *Diabetes* 66 1380–1390. 10.2337/db16-1181 28202580 PMC5399611

[B26] Matos Cunha L. C. Campelo Tavares M. Tierra Criollo C. J. Labanca L. Cardoso Dos Santos Couto Paz C. Resende Martins H. (2013). Contribution of galvanic vestibular stimulation for the diagnosis of HTLV-1-associated myelopathy/tropical spastic paraparesis. *J. Clin. Neurol.* 9:252. 10.3988/jcn.2013.9.4.252 24285967 PMC3840136

[B27] Matsugi A. Douchi S. Hasada R. Mori N. Okada Y. Yoshida N. (2020). Cerebellar repetitive transcranial magnetic stimulation and noisy galvanic vestibular stimulation change vestibulospinal function. *Front. Neurosci.* 14:388. 10.3389/fnins.2020.00388 32410952 PMC7198759

[B28] Matsugi A. Ueta Y. Oku K. Okuno K. Tamaru Y. Nomura S. (2017). Effect of gaze-stabilization exercises on vestibular function during postural control. *NeuroReport* 28 439–443. 10.1097/WNR.0000000000000776 28368883

[B29] Metz K. Matos I. C. Hari K. Bseis O. Afsharipour B. Lin S. (2023). Post-activation depression from primary afferent depolarization (PAD) produces extensor H-reflex suppression following flexor afferent conditioning. *J. Physiol.* 601 1925–1956. 10.1113/JP283706 36928599 PMC11064783

[B30] Mikhail Y. Charron J. Mac-Thiong J.-M. Barthélemy D. (2021). Assessing head acceleration to identify a motor threshold to galvanic vestibular stimulation. *J. Neurophysiol.* 125 2191–2205. 10.1152/jn.00254.2020 33881904

[B31] Nakamura J. Kita Y. Ikuno K. Kojima K. Okada Y. Shomoto K. (2015). Influence of the stimulus parameters of galvanic vestibular stimulation on unilateral spatial neglect. *NeuroReport* 26 462–466. 10.1097/WNR.0000000000000369 25875473

[B32] Nakamura J. Okada Y. Shiozaki T. Tanaka H. Ueta K. Ikuno K. (2021). Reliability and laterality of the soleus H-reflex following galvanic vestibular stimulation in healthy individuals. *Neurosci. Lett.* 755:135910. 10.1016/j.neulet.2021.135910 33910060

[B33] Nepveu J.-F. Mikhail Y. Pion C. H. Gossard J.-P. Barthélemy D. (2020). Assessment of vestibulocortical interactions during standing in healthy subjects. *PLoS One* 15:e0233843. 10.1371/journal.pone.0233843 32497147 PMC7272097

[B34] Nielsen J. Petersen N. Crone C. (1995). Changes in transmission across synapses of Ia afferents in spastic patients. *Brain* 118 995–1004. 10.1093/brain/118.4.995 7655894

[B35] Okada Y. Shiozaki T. Nakamura J. Azumi Y. Inazato M. Ono M. (2018). Influence of the intensity of galvanic vestibular stimulation and cutaneous stimulation on the soleus H-reflex in healthy individuals. *NeuroReport* 29 1135–1139. 10.1097/WNR.0000000000001086 29965870

[B36] Osler C. J. Reynolds R. F. (2012). Dynamic transformation of vestibular signals for orientation. *Exp. Brain Res.* 223 189–197. 10.1007/s00221-012-3250-1 22990288

[B37] Özyurt M. G. Topkara B. Şenocak B. S. Budan A. S. Yüce M. N. Türker K. S. (2020). Post-activation depression of primary afferents reevaluated in humans. *J. Electromyogr. Kinesiol.* 54:102460. 10.1016/j.jelekin.2020.102460 32905963

[B38] Peusner K. D. Shao M. Reddaway R. Hirsch J. C. (2012). Basic concepts in understanding recovery of function in vestibular reflex networks during vestibular compensation. *Front. Neur.* 3:17. 10.3389/fneur.2012.00017 22363316 PMC3282297

[B39] Pliego A. Vega R. Gómez R. Reyes-Lagos J. J. Soto E. (2021). A transient decrease in heart rate with unilateral and bilateral galvanic vestibular stimulation in healthy humans. *Eur. J. Neurosci.* 54 4670–4681. 10.1111/ejn.15338 34076918

[B40] Reimann H. Fettrow T. D. Thompson E. D. Agada P. McFadyen B. J. Jeka J. J. (2017). Complementary mechanisms for upright balance during walking. *PLoS One* 12:e0172215. 10.1371/journal.pone.0172215 28234936 PMC5325219

[B41] Salinas L. F. Trujillo-Condes V. E. Tecuatl C. Delgado-Lezama R. Cuellar C. A. (2022). Impaired rate-dependent depression of the H-reflex in type-2 diabetes, prediabetes, overweight and obesity: A cross-sectional study. *Medicine* 101 e31046. 10.1097/MD.0000000000031046 36316945 PMC9622671

[B42] Sayenko D. G. Atkinson D. A. Mink A. M. Gurley K. M. Edgerton V. R. Harkema S. J. (2018). Vestibulospinal and corticospinal modulation of lumbosacral network excitability in human subjects. *Front. Physiol.* 9:1746. 10.3389/fphys.2018.01746 30574093 PMC6291495

[B43] Schniepp R. Boerner J. C. Decker J. Jahn K. Brandt T. Wuehr M. (2018). Noisy vestibular stimulation improves vestibulospinal function in patients with bilateral vestibulopathy. *J. Neurol.* 265 57–62. 10.1007/s00415-018-8814-y 29508134

[B44] Shinoda Y. Ohgaki T. Futami T. (1986). The morphology of single lateral vestibulospinal tract axons in the lower cervical spinal cord of the cat. *J. Comp. Neurol.* 249 226–241. 10.1002/cne.902490208 3734158

[B45] Sprenger A. Spliethoff P. Rother M. Machner B. Helmchen C. (2020). Effects of perceptible and imperceptible galvanic vestibular stimulation on the postural control of patients with bilateral vestibulopathy. *J. Neurol.* 267 2383–2397. 10.1007/s00415-020-09852-x 32350649

[B46] Sugita A. Bai R. Imagawa M. Sato H. Sasaki M. Kitajima N. (2004). Properties of horizontal semicircular canal nerve-activated vestibulospinal neurons in cats. *Exp. Brain Res.* 156 478–486. 10.1007/s00221-003-1805-x 15007578

[B47] Suzuki S. Nakajima T. Irie S. Ariyasu R. Komiyama T. Ohki Y. (2017). Vestibular stimulation-induced facilitation of cervical premotoneuronal systems in humans. *PLoS One* 12:e0175131. 10.1371/journal.pone.0175131 28388686 PMC5384664

[B48] Tanaka H. Nakamura J. Siozaki T. Ueta K. Morioka S. Shomoto K. (2021). Posture influences on vestibulospinal tract excitability. *Exp. Brain Res.* 239 997–1007. 10.1007/s00221-021-06033-8 33479869

[B49] Tax C. M. W. Bom A. P. Taylor R. L. Todd N. Cho K.-K. J. Fitzpatrick R. C. (2013). The galvanic whole-body sway response in health and disease. *Clin. Neurophysiol.* 124 2036–2045. 10.1016/j.clinph.2012.12.041 23849702

[B50] Wardman D. L. Day B. L. Fitzpatrick R. C. (2003). Position and velocity responses to galvanic vestibular stimulation in human subjects during standing. *J. Physiol.* 547 293–299. 10.1113/jphysiol.2002.030767 12562970 PMC2342615

[B51] Welgampola M. S. Ramsay E. Gleeson M. J. Day B. L. (2013). Asymmetry of balance responses to monaural galvanic vestibular stimulation in subjects with vestibular schwannoma. *Clin. Neurophysiol.* 124 1835–1839. 10.1016/j.clinph.2013.03.015 23643313 PMC3745707

[B52] Worthington A. Kalteniece A. Ferdousi M. D’Onofrio L. Dhage S. Azmi S. (2021). Optimal utility of H-Reflex RDD as a biomarker of spinal disinhibition in painful and painless diabetic neuropathy. *Diagnostics* 11:1247. 10.3390/diagnostics11071247 34359330 PMC8306975

[B53] Wuehr M. Boerner J. C. Pradhan C. Decker J. Jahn K. Brandt T. (2018). Stochastic resonance in the human vestibular system – Noise-induced facilitation of vestibulospinal reflexes. *Brain Stimul.* 11 261–263. 10.1016/j.brs.2017.10.016 29100928

